# Dissolved Organic Phosphorus Production during Simulated Phytoplankton Blooms in a Coastal Upwelling System

**DOI:** 10.3389/fmicb.2012.00274

**Published:** 2012-08-06

**Authors:** K. C. Ruttenberg, S. T. Dyhrman

**Affiliations:** ^1^Department of Oceanography, School of Ocean and Earth Science and Technology, University of HawaiiHonolulu, HI, USA; ^2^Department of Geology and Geophysics, School of Ocean and Earth Science and Technology, University of HawaiiHonolulu, HI, USA; ^3^Biology Department, Woods Hole Oceanographic InstitutionWoods Hole, HI, USA

**Keywords:** phosphorus, dissolved organic phosphorus, nutrient, coastal ocean, upwelling, alkaline phosphatase activity, phosphomonoester

## Abstract

Dissolved organic phosphorus (DOP) is increasingly recognized as an important phosphorus source to marine primary producers. Despite its importance, the production rate and fate of DOP is poorly understood. In this study, patterns of DOP production were evaluated by tracking the evolution of DOP during simulated phytoplankton blooms initiated with nutrient amended surface waters, relative to controls, from the Oregon (USA) coastal upwelling system. Nitrogen (N) and phosphorus (P) additions were used to decouple DOP production and hydrolysis by inducing or repressing, respectively, community alkaline phosphatase activity. In order to examine the progression of nutrient uptake and DOP production under upwelling versus relaxation conditions, two experiments were initiated with waters collected during upwelling events, and two with waters collected during relaxation events. Maximum [under (+P) conditions] and minimum [under (+N) conditions] DOP production rates were calculated and applied to *in situ* DOP levels to evaluate which end-member rate most closely approximates the *in situ* DOP production rate at the four study sites in this coastal system. Increases in DOP concentration occurred by day-5 in control treatments in all experiments. N treatments displayed increased chlorophyll *a*, increased alkaline phosphatase activity, and yielded lower net DOP production rates relative to controls, suggesting that DOP levels were depressed as a consequence of increased hydrolysis of bioavailable DOP substrates. Phosphorus additions resulted in a significant net production of DOP at all stations, but no increase in chlorophyll *a* relative to control treatments. The contrasting patterns in DOP production between treatments suggests that changes in the ambient dissolved inorganic nitrogen:dissolved inorganic phosphorus (DIN:DIP) ratio could exert profound control over DOP production rates in this system. Patterns of DOP production across the different experiments also suggest that bathymetry-driven differences in water residence times can influence DOP cycling. Taken together, these factors may impact the potential export of DOP to offshore ecosystems.

## Introduction

Phosphorus (P) is an essential nutrient, required for many diverse biological functions, such as membrane synthesis and energy transfer. Although dissolved inorganic phosphorus (DIP) distribution patterns and processes have been widely studied in marine systems for decades, the dissolved organic phosphorus (DOP) pool has received little attention until relatively recently. DOP represents a major reservoir of dissolved phosphorus in surface waters of both oligotrophic systems, where DOP typically comprises >75% of total dissolved P (TDP) (Jackson and Williams, 1985; Abell et al., 2000; Benitez-Nelson, 2000; Wu et al., 2000; Karl and Björkman, 2002), and coastal marine systems where DOP can comprise up to ≈90% TDP (Thingstad et al., 1993; Furnas and Mitchell, 1999; Monaghan and Ruttenberg, 1999; Karl and Björkman, 2002; Ruttenberg and Dyhrman, 2005).

Over the past decade, DOP has been increasingly recognized as a critical driver of oceanic biological production and ecosystem structure and function (Karl and Björkman, 2002; Dyhrman et al., 2006, 2007; Paytan and McLaughlin, 2007; Mather et al., 2008; Lomas et al., 2010). DOP utilization by phytoplankton is enabled by the activities of hydrolytic enzymes such as alkaline phosphatase (Dyhrman and Palenik, 1999) and C-P lyase (Dyhrman et al., 2006), which release bioavailable DIP from organic substrates. These hydrolytic enzymes are typically up-regulated when DIP concentrations drop below critical threshold levels (Dyhrman and Palenik, 1999; Dyhrman et al., 2006). While a few studies have shown species-specific abilities to produce the enzymes required for hydrolysis of phosphate from phosphonates (Dyhrman et al., 2006) and diesters (Dyhrman et al., 2012), the alkaline phosphatase enzyme that hydrolyzes phosphomonoesters is known to be widespread (Dyhrman et al., 2007), and likely plays a major role in driving the hydrolysis of the ester pool, which appears to dominate DOP (Young and Ingall, 2010) in marine systems.

Despite an increasing number of studies focused on the oceanic DOP pool, the dynamics of DOP production and consumption processes remain poorly constrained. While numerous studies have demonstrated an accumulation of dissolved organic matter (DOM) after phytoplankton blooms and over the course of the growing season in a variety of marine systems (Ittekkot et al., 1981; Bronk et al., 1994; Williams, 1995; Wetz and Wheeler, 2003), and the fate of DOC and DON within this pool has been highly scrutinized (Kirchman et al., 1991; Amon and Benner, 1994, 1996; Emerson and Hayward, 1995; Letelier and Karl, 1996; Thingstad et al., 1997; Karl et al., 1998; Abell et al., 2000; Wetz et al., 2008), less attention has been focused on DOP. Studies that have focused on DOP cycling commonly use ^32^P or ^33^P as tracers for P cycling, or single compounds as “model” compounds for DOP (see Karl and Björkman, 2002 for review of numerous studies), rather than examining the evolution of the bulk, *in situ* DOP pool. Exceptions include Abell et al. (2000) and Hopkinson et al. (2002), who estimated remineralization rates of bulk DOP (along with DOC and DON), but did not account for simultaneous DOP production that may have been occurring. It is particularly crucial to examine DOP production in coastal systems because the lateral supply of DOP from continental margins to the ocean gyres may be significant. For example Mahaffey et al. (2004) suggest that the lateral supply of DOP is a factor of two to three times more important than that of semi-labile DON to the eastern North Atlantic, and speculate (as have others: e.g., Lomas et al., 2010) that lateral supply of DOP could support primary production in the North Atlantic Subtropical Gyre.

**Figure 1:**
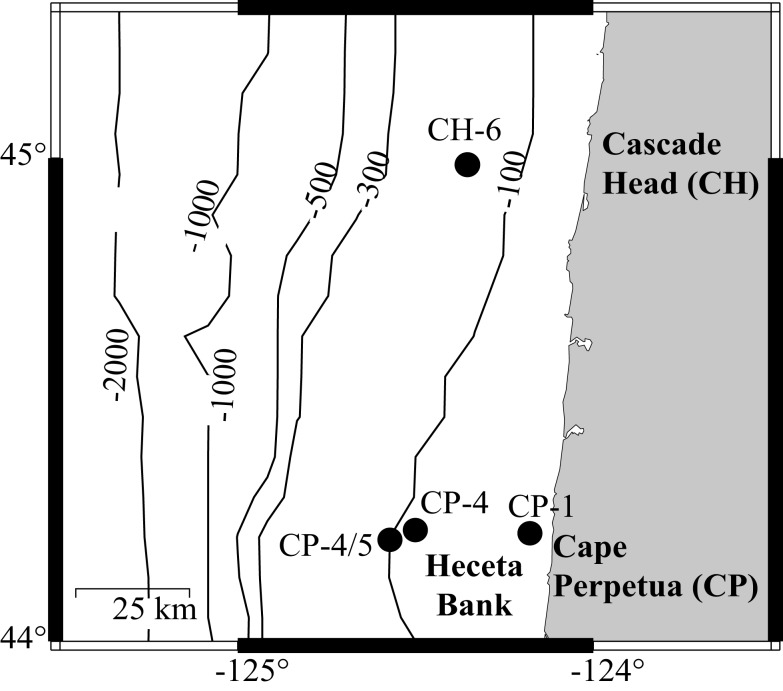
**Field sites where incubation waters were collected, one on the CH-line (CH-6), and three on the Cape Perpetua Line (CP-1, CP-4, and CP-4/5)**.

In the study described here, we examine the dynamics of DOP production in simulated phytoplankton blooms in waters from the coastal Oregon upwelling region. Although biological productivity in coastal upwelling systems is traditionally viewed as driven by N limitation, recent studies provide strong evidence of a potentially important role for DOP in supporting primary production (Monaghan and Ruttenberg, 1999; Labry et al., 2002; Vidal et al., 2003; Ruttenberg and Dyhrman, 2005; Dyhrman and Ruttenberg, 2006; Spitz et al., 2010). Five-day ship-board incubation experiments were conducted with surface waters taken within the Coastal Ocean Processes (CoOP) COAST study grid, a region that has been extensively studied for nitrogen and carbon dynamics, phytoplankton productivity, and physical processes by the CoOP-COAST program and prior studies (Barth and Wheeler, 2005). Four experiments were initiated using surface waters from mid-shelf sites (Figure 1); two with waters collected during upwelling events, and two with waters collected during relaxation events, so that patterns of DOP production under upwelling versus relaxation conditions could be examined. In order to decouple potential DOP production from consumption, DOP was assayed in grow-out experiments with nutrient amendments that forced maximum and minimum DOP consumption [(+N) and (+P) treatments, respectively], by taking advantage of the fact that alkaline phosphatase activity (APA) is regulated by phosphate, meaning that it is induced under low DIP conditions (+N), and suppressed under high DIP conditions (+P). Both nutrient addition treatments were examined relative to controls (no additions). Results of these experiments allow us to identify factors that potentially control DOP production rates under different environmental conditions.

## Materials and Methods

### Study site

The sampling grid occupied by the CoOP-COAST program was the site of a 5-year multi-disciplinary project aimed at studying seasonal patterns of circulation, biology, and chemistry in the upwelling-dominated Oregon coastal ocean (see Barth and Wheeler, 2005, for project overview and summary). Located off the Oregon Coast, extending from *ca*. 45.2–43.7°N, and from shallow inshore stations (*ca*. 30 m water depth) to offshore stations (*ca*. 1000 m water depth), this coastal area is characterized by frequent upwelling-relaxation events during spring and summer (Barth et al., 2005; Castelao and Barth, 2005). High-resolution sampling was conducted during three cruises (spring and summer 2001, winter 2003) to evaluate the dependence of biological and biogeochemical parameters on upwelling, downwelling, along-shore, and cross-shelf transport. The incubation experiments described in this study were conducted during the summer 2001 cruise.

### Incubation experiments

Surface water samples from four sites within the COAST sampling grid were obtained for incubation studies (Figure 1; Table 1): two collected during upwelling (CP-1, CP-4/5) and two during relaxation conditions (CH-6 and CP-4). Sites CP-1 and CP-4/5 were defined as upwelling sites based on colder surface temperatures (11.1 and 12.5°C, respectively) at the time of collection, while the two relaxation sites (CH-6 and CP-4) had temperatures in excess of 13°C. Initial chlorophyll *a* (chl *a*) and dissolved P concentrations are also consistent with these definitions (Ruttenberg and Dyhrman, 2005). Discrete large volume samples were collected at these sites from a continuous pump-profiler (Hales et al., 2005) by arresting the profiler at the pre-determined sampling depth (≈5 m below surface), and diverting the sample water stream into 20-L carboys. Water from the collection carboys was passed through a 100 μm Nitex screen to remove larger zooplankton, and this screened water collected in a second 20-L carboy. Carboys were acid (10% HCl) cleaned and rinsed three times with sample water prior to collection of water for experiments. After first gently inverting the carboy to homogenize, water was collected for analysis of initial condition biogeochemical parameters, and then was partitioned into nine acid (10% HCl) cleaned, 4-L polycarbonate bottles, after first rinsing bottles three times with sample water. Triplicate incubation bottles were spiked with 30 μM K_2_HPO_4_ (+P), 100 μM KNO_3_ (+N), or left unspiked (control), respectively. The nutrient amendments were chosen to be roughly 10× the upwelling average nutrient concentrations (∼3 μM DIP and ∼13 μM DIN). Although high, these spike levels were necessary to avoid substantial drawdown during the long (5 day) incubation time course. The extended incubation time was required to sustain the effect of nutrient amendments for a sufficiently long period to enable observation of changes in the DOP pool. Incubation bottles were placed in an incubating bath plumbed with continuously circulating surface seawater to maintain them at surface water temperatures. The bath, located on the fantail of the ship, was covered with Nitex screen such that light levels approximated those at 5 m water depth.

**Table 1:** **Station information**.

Station #	Sampling date	Latitude (N)	Longitude (W)	Water depth (m)	Sampling depth (m)	Salinity	Temp (°C)
**UPWELLING CONDITIONS**							
CP-1	8/13/01	44°13.50′	124°08.80′	35	5	33.44	11.1
CP-4/5^a^	8/17/01	44°13.49′	124°36.75′	109	3	32.72	12.5
**RELAXATION CONDITIONS**							
CH-6	8/9/01	45°00.29′	124°21.35′	181	5	32.30	14.2
CP-4	8/16/01	44°13.50′	124°28.10′	102	5	32.43	13.2

*^a^Station CP-4/5 is identified as CP-6 in Dyhrman and Ruttenberg (2006)*.

All bottles were removed from the bath on day-3 and -5 of the incubation, brought into the shipboard laboratory, and sub-sampled for analysis of TDP, DIP, chl *a*, and APA (note: APA was only measured on day-5 due to sample volume limitations). Immediately after sub-sampling on day-3, bottles were capped and returned to the shipboard incubating bath. Filtration of sub-samples commenced as soon as possible after collection, usually within ten minutes. After gently inverting sub-sample bottles to homogenize, splits were filtered through 0.2 μm polypropylene filters for analysis of TDP and DIP. Samples were vacuum filtered using Nalgene^®^ polysulfone filtration apparatus at low pressure (≤5 psi) to prevent cell lysis (e.g., Bidigare, 1991; Karl et al., 1991; Matrai, 1991; Mopper and Furton, 1991). Filtration apparatus were acid cleaned (10% HCl) prior to initial use; between samples the upper reservoir was rinsed with unfiltered sample water, and the lower reservoir with the first 50-mL of filtrate to pass through the filter. Filtrates were collected in acid cleaned (10% HCl) polypropylene bottles, acidified to pH 1 with trace-metal clean HCl, and stored refrigerated until analysis (Monaghan and Ruttenberg, 1999). Polycarbonate filters were soaked for ≥48 h in 10% HCl, removing and replacing acid at least three times during soaking, and then were soaked for ≥24 h in acidified methanol to remove organic wetting agents. All 0.2 μm filters were stored in 10% HCl in acid-cleaned glass jars, and rinsed copiously with Milli-Q water just prior to use. Filtration blanks for dissolved P were below detection. For whole community APA, 500-mL of sample was filtered through a 0.2 μm polycarbonate filter; filtration and filter storage were as described above for dissolved P.

### Analytical methods

Total Dissolved Phosphorus was determined on 0.2 μm filtrates using the high-temperature ashing/hydrolysis method of Solórzano and Sharp (1980), as modified by Monaghan and Ruttenberg (1999). Soluble Reactive Phosphorus (SRP) was determined by the standard phosphomolybdate blue method according to Koroleff (1983). Analytical precision for TDP and SRP is ±0.02 and ±0.01 μM, respectively (Dyhrman and Ruttenberg, 2006). We make the assumption that SRP is equivalent to DIP (see Ruttenberg and Dyhrman, 2005 for justification). DOP is calculated as the difference between TDP and SRP, both of which are measured quantities with their own associated analytical uncertainties. The uncertainty associated with the derived DOP values was calculated by propagating the errors in TDP and DIP, as previously described in Ruttenberg and Dyhrman (2005). Because samples were acidified (pH 1) immediately after collection and stored for a number of weeks prior to analysis, short chain polyphosphates would have hydrolyzed to DIP prior to analysis (Monaghan and Ruttenberg, 1999), such that DOP concentrations reported here should not include polyphosphate. Analysis of whole community APA was performed on cell concentrates as described by Ruttenberg and Dyhrman (2005) and Dyhrman and Ruttenberg (2006). Specifically, DiFMUP was used as a DOP analog at saturating concentrations in order to obtain maximum hydrolysis rates, or *V*_max_ values. It was not realistic to perform kinetics experiments on incubation samples due to sample volume limitations, and so *in situ* rates were not determined. Chl *a* was measured on whole water samples collected onto GF/F filters using a standard 95% methanol extraction; see Wetz and Wheeler (2005) for detailed description of the assay.

### Derived rates and statistics

Rates of change of DIP and DOP concentrations in each treatment, calculated by subtracting concentrations on day-5 from initial concentrations, allow us to evaluate rates of DIP drawdown and DOP production for each site and for each treatment over the 5-day incubation time course. Both raw rates (change in concentration over time) and biomass-normalized rates (raw rates divided by chl *a* concentrations measured on day-5) were calculated. Rates were calculated for each replicate separately, and the mean over the three replicates for each treatment is reported in Table 3 along with the standard deviation about the mean (*n* = 3). An estimate of uncertainty for all derived values is included and was calculated as per the notes in the associated tables. Where reported, statistical significance (*p* < 0.05) was determined on pair-wise comparisons using a *t*-test.

## Results

### Chl *a*, dissolved P concentrations, and alkaline phosphatase activities

**Figure 2:**
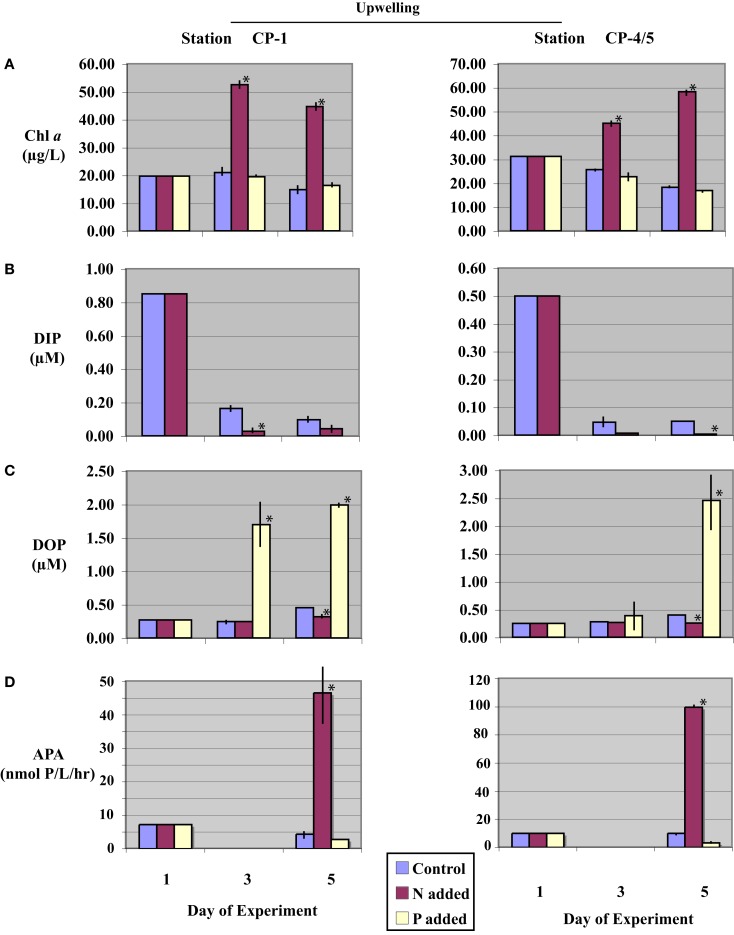
**Time-course incubation results initiated with waters collected during an upwelling event (stations CP-1 and CP-4/5) and incubated with nitrogen (+N), phosphate (+P), or no addition (control)**. Initial (day-1) values reflect *in situ* values, days-3 and -5 reflect concentrations or activities on subsequent days of the incubation. **(A)** Chl *a* concentrations; **(B)** Dissolved Inorganic Phosphorus (DIP) concentrations (data from (+P) treatments are not shown, see text); **(C)** Dissolved Organic Phosphorus (DOP) concentrations; and **(D)** Alkaline phosphatase activity (APA), APA was not assayed on day-3. Error bars represent the standard deviation of measurements from triplicate incubation bottles (where not shown, error bars were too small to resolve on the scale of the figure); replicates were not available for initial (day-1) samples. Asterisks (*) denote values significantly different from control (*p* < 0.05).

**Figure 3:**
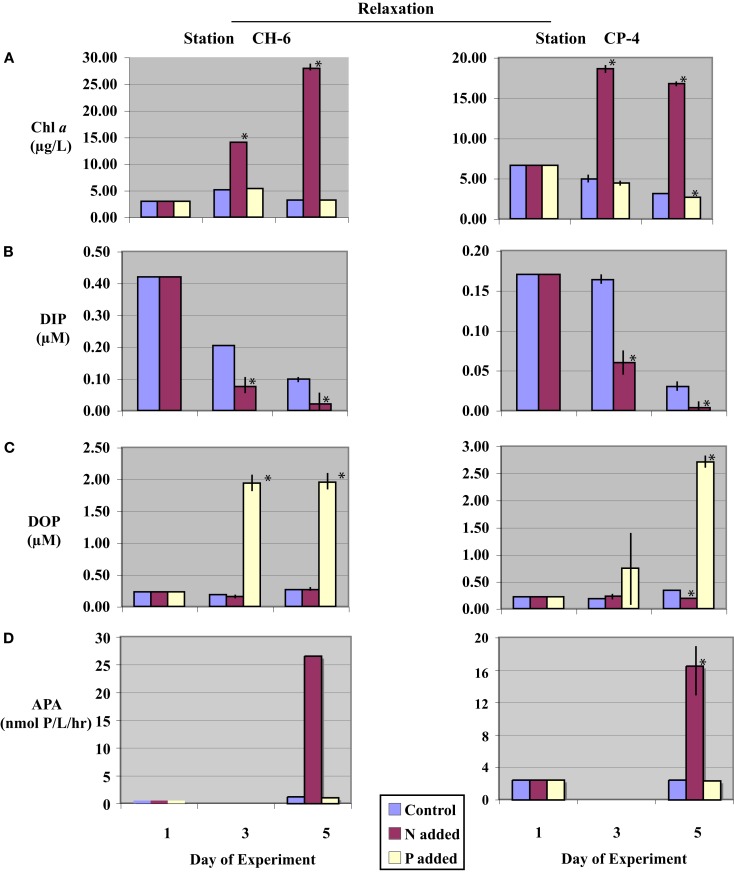
**Time-course incubation results initiated with waters collected during a relaxation event (stations CH-6 and CP-4) and incubated with nitrogen (+N), phosphate (+P), or no addition (control)**. Initial (day-1) values reflect *in situ* values, days-3 and -5 reflect concentrations or activities on subsequent days of the incubation. **(A)** Chl *a* concentrations; **(B)** Dissolved Inorganic Phosphorus (DIP) concentrations (data from (+P) treatments are not shown, see text); **(C)** Dissolved Organic Phosphorus (DOP) concentrations; and **(D)** Alkaline phosphatase activity (APA), APA was not assayed on day-3. Error bars represent the standard deviation of measurements from triplicate incubation bottles (where not shown, error bars were too small to resolve on the scale of the figure); replicates were not available for initial (day-1) samples. Asterisks (*) denote values significantly different from controls (*p* < 0.05).

Initial chl *a* concentrations collected under upwelling conditions (stations CP-1 and CP-4/5) were higher than those observed under relaxation conditions (stations CH-6 and CP-4; Table 2). Chl *a* was significantly increased (*p* < 0.05) above initial conditions over the time-course for all (+N) treatments (Figures 2A and 3A). The increase was monotonic in Station CP-4/5 and CH-6 incubations, whereas in Station CP-1 and CP-4 incubations, day-3 concentrations were higher than day-5. In control and (+P) treatments, in contrast, chl *a* concentrations decreased over the time-course such that on day-5, chl *a* levels in (+P) treatments were not significantly different (*p* > 0.05) from controls (Table 3).

**Table 2:** **Initial (*in situ*) conditions**.

Station #	Chl *a*^a^ (μg/L)	Stdev^a^ (μg/L)	DIP^b^ (μM)	TDP^b^ (μM)	DOP^b^ (μM)	APA (nmol P/L/h)	Specific^c^ APA (nmol P/μg Chl *a*/h)
**UPWELLING CONDITIONS**							
CP-1	19.63	0.46	0.85	1.12	0.27	2.926	0.148
CP-4/5	31.17	n/a^d^	0.50	0.74	0.25	1.680	0.054
**RELAXATION CONDITIONS**							
CH-6	2.95	0.05	0.42	0.65	0.23	0.296	0.010
CP-4	6.62	0.19	0.17	0.39	0.22	3.184	0.048

*^a^Replicate filters (*n* = 3) were collected for Chl *a*; Stdev indicates standard deviation over triplicate filters except for CP-4/5, where replicate filters were not collected*.

*^b^Because replicates of initial dissolved P were not collected, precision is estimated from analytical precision determinations for DIP (±0.01 μM) and TDP (±0.02 μM); precision of DOP (±0.02 μM) is estimated from error propagation as described in Ruttenberg and Dyhrman (2005)*.

*^c^Specific APA is the raw APA divided by the initial Chl *a* concentration for each site. It is also referred to as the biomass-normalized APA*.

*^d^n/a, not available*.

**Table 3:** **Mean^a^ DIP drawdown and DOP production rates calculated over 5-day incubation experiments**.

Station	Treatment		DIP-drawdown (nmol P/L/h)	Biomass-normalized DIP-drawdown^b^ (nmol P/μg Chl *a*/h)	DOP-production (nmol P/L/h)	Biomass-normalized DOP-production^b^ (nmol P/μg Chl *a*/h)	Day-5 Chl *a* (μg/L)
**UPWELLING CONDITIONS**							
CP-1	Control	Mean	−6.28	−0.44	1.53	0.11	14.75
		Stdev	0.21	0.08	0.13	0.02	3.32
	(+N)	Mean	−6.72	−0.15	0.39	0.01	44.63
		Stdev	0.24	0.01	0.46	0.01	3.49
		^c^*p* (N versus control)	*0.073*	*0.004*	*0.014*	*0.001*	<*0.001*
	(+P)	Mean	−3.78	−0.22	14.33	0.88	16.28
		Stdev	4.36	0.25	0.58	0.06	1.74
		*p* (P versus control)	*0.377*	*0.232*	*0.014*	<*0.001*	*0.518*
CP-4/5	Control	Mean	−3.75	−0.21	1.22	0.07	18.23
		Stdev	0.08	0.02	0.31	0.02	1.10
	(+N)	Mean	−4.17	−0.07	0.06	0.00	58.27
		Stdev	0.00	0.00	0.35	0.01	1.95
		*p* (N versus control)	*0.001*	*0.003*	*0.013*	*0.006*	<*0.001*
	(+P)	Mean	−4.03	−0.23	18.39	1.07	16.91
		Stdev	4.22	0.24	9.11	0.49	0.78
		*p* (P versus control)	*0.915*	*0.869*	*0.031*	*0.019*	*0.166*
**RELAXATION CONDITIONS**							
CH-6	Control	Mean	−2.68	−0.84	0.27	0.08	3.22
		Stdev	0.08	0.08	0.22	0.07	0.20
	(+N)	Mean	−3.29	−0.12	0.29	−0.01	27.98
		Stdev	0.36	0.01	0.44	0.02	1.88
		*p* (N versus control)	*0.046*	<*0.001*	*0.946*	*0.341*	<*0.001*
	(+P)	Mean	−0.71	−0.21	14.35	4.50	3.18
		Stdev	2.14	0.66	2.15	0.45	0.18
		*p* (P versus control)	*0.186*	*0.176*	<*0.001*	<*0.001*	*0.821*
CP-4	Control	Mean	−1.19	−0.38	1.00	0.32	3.13
		Stdev	0.05	0.03	0.25	0.09	0.20
	(+N)	Mean	−1.39	−0.08	−0.25	−0.01	16.76
		Stdev	0.05	0.01	0.36	0.02	0.41
		*p* (N versus Control)	*0.008*	<*0.001*	*0.008*	*0.004*	<*0.001*
	(+P)	Mean	−3.83	−1.50	20.75	7.80	2.66
		Stdev	5.55	2.11	2.02	0.36	0.14
		*p* (P versus control)	*0.008*	*0.410*	<*0.001*	<*0.001*	*0.029*

*^a^Mean and Standard Deviation (Stdev) for each treatment was calculated over triplicate bottles*.

*^b^Biomass-normalized rates were calculated by dividing raw rates by Chl *a* concentrations measured on day-5*.

*^c^*p*-values were calculated using a *t*-test comparing each nutrient addition treatment [(+N) and (+P), respectively] to controls for each station; values <0.05 indicate that the treatment is considered significantly different from the control*.

Initial DIP concentrations were highest in the upwelling stations, ranging between 0.50 and 0.85 μM in upwelling and between 0.17 and 0.42 μM in relaxation stations (Table 2), but did not vary systematically as a function of upwelling versus relaxation conditions. DIP concentrations were significantly lower (*p* < 0.05) in (+N) treatments relative to the Control treatments for all experiments by day-5 (Figures 2B and 3B). DIP was higher in (+P) treatments than in the Controls at day-5 in all experiments (data not shown).

Initial DOP concentrations ranged between 0.22 and 0.27 μM across all stations (Table 2), with no major difference between the upwelling and relaxation sites. By day-5, DOP concentrations in (+N) treatments were significantly (*p* < 0.05) lower than controls in both upwelling (CP-1 and CP-4/5, Figure 2C) and one relaxation (CP-4, Figure 3C) experiment. The other relaxation site (Station CH-6, Figure 3C) was the only experiment in which day-5 DOP concentrations in (+N) treatments were not significantly depressed relative to the control (*p* > 0.05). DOP became significantly (*p* < 0.05) elevated in the (+P) treatments relative to controls by day-5 in all experiments (Figures 2C and 3C).

Alkaline phosphatase activity was present in all initial samples (Table 2), and neither APA nor specific APA varied systematically between upwelling and relaxation sites. All experiments had significantly higher (*p* < 0.05) APA in (+N) treatments relative to controls (Figures 2D and 3D). Day-5 APA in (+P) treatments was lower than initial conditions in all experiments except CH-6, and APA was significantly lower (*p* < 0.05) in (+P) relative to the control for CP-4 (Figures 2D and 3D).

### Dissolved inorganic P drawdown and dissolved organic P production rates

Negative rates of DIP concentration change, indicating DIP drawdown, were observed at all stations in all treatments by day-5 (Table 3). Rates of DIP drawdown were significantly larger (*p* < 0.05) in (+N) treatments relative to controls in three of the four incubations, with station CP-1 the exception (Table 3). Rates of change in (+P) treatments at all sites were negative, indicating drawdown, but were based on differences in concentration that were small relative to initial elevated DIP levels, making it difficult to confidently resolve them within the large variance about the mean rate (Table 3).

Positive rates of DOP concentration change, indicating an excess of DOP production over DOP hydrolysis, were observed at all stations in all treatments on day-5, excepting the (+N) treatment for station CP-4. Rates of DOP change in (+P) treatments at all sites were strongly positive and significantly (*p* < 0.05) higher than Controls (Table 3). It is important to note that any DOP production observed reflects net production. In other words, the DOP concentration present at any given time during the incubation reflects a balance between DOP production (which can include passive exudation or excretion, as well as *de novo* production) and DOP consumption (via hydrolysis or direct uptake). Rates reported in Table 3 are thus net rates of DOP production, and should be considered as minimum estimates across all treatments. Therefore, while we use the term ‘DOP Production’ in this paper in order to expedite and simplify discussion, we acknowledge that observed DOP accumulation in our experiments reflects apparent production only, and likely occurs via a number of distinct pathways.

DOP production rates calculated for the different experimental treatments (Table 3) were applied to initial DOP concentrations at each site to calculate the maximum (+P) and minimum (+N) predicted net DOP (Table 4) expected at the end of the 5-day simulated phytoplankton blooms. Because all rates are calculated from day-5, treatments with DOP concentration peaking earlier than day-5 (CH-6, CP-1) underestimate production rates. In effect, therefore, rates reported in Table 4 for these treatments should be considered as minimum estimates across both (+N) and (+P) treatments. The maximum predicted net DOP was higher than observed values at all stations (Table 4). In contrast, the minimum predicted net DOP was lower than observed values at all sites, with the exception of CH-6, where predicted values were indistinguishable from observed values (Table 4). Note that uncertainties associated with rates and predicted DOP concentrations are higher in (+N) treatments than (+P) treatments because the magnitude of DOP of change in (+N) treatments is small to negligible, and thus the apparent rate of DOP production derived from these small changes in DOP concentration is low.

**Table 4:** **Prediction of the *in situ* balance between net DOP production and DOP consumption during 5-day, simulated phytoplankton blooms at four stations on the Oregon Coast^a^**.

Conditions	Upwelling		Relaxation	
Station	CP-1	CP-4/5	CH-6	CP-4
Initial (*in situ*) [DOP] μM^b^	0.27 (0.02)	0.25 (0.02)	0.23 (0.02)	0.22 (0.02)
Observed [DOP]_control_ (μM) @ day-5^c^	0.45 (0.02)	0.40 (0.04)	0.26 (0.03)	0.34 (0.03)
**(+P) TREATMENT**				
Maximum net DOP production rate (nmol P/L/h)^c^^,^^d^	14.33 (0.58)	18.39 (9.11)	14.35 (2.15)	20.75 (2.02)
Predicted net DOP accumulated over 5-day incubation (μM)^e^^,^^f^	1.72 (0.07)	2.21 (1.09)	1.72 (0.26)	2.49 (0.24)
Maximum predicted net [DOP] concentration at day-5 (μM)^g^^,^^h^	1.99 (0.07)	2.46 (1.09)	1.95 (0.26)	2.71 (0.24)
Difference between Maximum predicted net and observed [DOP]_control_ concentration @ day-5 (μM)^i^	1.54 (0.07)	2.06 (1.09)	1.69 (0.26)	2.37 (0.24)
Observations	[DOP]_predicted_ > [DOP]_obsd_	[DOP]_predicted_ > [DOP]_obsd_	[DOP]_predicted_ > [DOP]_obsd_	[DOP]_predicted_ > [DOP]_obsd_
Implications for [DOP]	Production > consumption	Production > consumption	Production > consumption	Production > consumption
**(+N) TREATMENT**				
Minimum net DOP production rate (nmol P/L/h)^c^^,^^d^	0.39 (0.46)	0.06 (0.35)	0.29 (0.44)	−0.25 (0.36)
Predicted net DOP accumulated over 5-day incubation (μM)^e^^,^^f^	0.05 (0.06)	0.01 (0.06)	0.04 (0.06)	−0.03 (0.04)
Minimum predicted net [DOP] concentration at day-5 (μM)^g^^,^^h^	0.32 (0.06)	0.26 (0.06)	0.27 (0.06)	0.19 (0.04)
Difference between Minimum predicted net and observed [DOP]_control_ concentration @ day-5 (μM)^i^	−0.13 (0.06)	−0.14 (0.07)	0.01 (0.07)	−0.15 (0.05)
Observations	[DOP]_predicted_ > [DOP]_obsd_	[DOP]_predicted_ > [DOP]_obsd_	[DOP]_predicted_ ≈ [DOP]_obsd_	[DOP]_predicted_ > [DOP]_obsd_
Implications for [DOP]	Consumption > production	Consumption > production	Consumption ≈ production	Consumption > production

*^a^Values in parentheses are uncertainties, estimated as described in the Section “Materials and Methods” and according to the Table notes for each parameter*.

*^b^Uncertainty in Initial DOP is the analytical uncertainty (±0.02 μM), as described in Note *b* to Table 2*.

*^c^Uncertainties for Observed day-5 DOP concentrations and for net DOP Production Rates are estimated as the standard deviation about the mean calculated over concentrations and rates determined on triplicate bottles*.

*^d^Rates are from Table 3*.

*^e^Predicted net [DOP] accumulation over the 5-day incubation is calculated as rate (nM-DOP/h) × 120 h = [DOP] (nM), where 5-days equates to 120 h*.

*^f^Uncertainty in Predicted net [DOP] production over the 5-day incubation is calculated according the standard error propagation formulation: ({square root [(Δ_rate_/Rate)^2^ + (Δ_time_/Time)^2^]} × [DOP]_predicted day-5 net [DOP]_), where Δ_rate_ is calculated as described in Note c, above, and Δ_time_ (the uncertainty in time of sample collection during the time course incubation) is ±0.5 h*.

*^g^Predicted net [DOP] concentration is calculated as the initial (*in situ*) [DOP] + {predicted day-5 net [DOP]}. Concentrations are identified as ‘net’ owing to the fact that the rates are calculated based on subtraction of initial from day-5 DOP concentrations, and the latter reflect both production and consumption that occurred over the 5-day incubation time-course; see text for further discussion*.

*^h^Uncertainty in the Predicted Net [DOP] concentration at day-5 is calculated by propagating the error in the initial [DOP] and the Predicted net [DOP] production at day-5, according to the following standard error propagation formulation: square root {(Δ_initial [DOP]_)^2^ + (Δ_predicted net [DOP] production at day-5_)^2^}, where Δ_initial [DOP]_ and Δ_predicted net [DOP] production at day-5_ are calculated as described in Notes b and f, above*.

*^i^Uncertainty in the difference between Predicted net [DOP] and Observed [DOP]_control_ @ day-5 is calculated by propagating the error associated with these two quantities according to the standard error propagation formulation: square root {(Δ_Predicted net [DOP] @ day-5_)^2^ + (Δ_Observed control [DOP] @ day-5_)^2^}, where (Δ_Predicted net [DOP] concentration @ day-5_), and (Δ_Observed control [DOP] @ day-5_) are calculated as described in notes h and c, respectively*.

## Discussion

In coastal upwelling systems, DOM production and release by phytoplankton is increasingly recognized as an important component of oceanic carbon and nitrogen cycling (Hansell and Carlson, 1998; Alvarez-Salgado et al., 2001; Wetz and Wheeler, 2007; Wetz et al., 2008). Unlike POM, which is subject to export, the fate of autochthonous DOM is controlled mainly by advective processes. As such, productive coastal upwelling systems have been recognized as a potentially important but underappreciated source of DON and DOC to oligotrophic regions of the ocean. The production and release of DOP in these systems, however, has been largely unexplored. The experimental work described here was designed to begin to address this knowledge gap by tracking DOP production during simulated phytoplankton blooms initiated in waters collected during the summer upwelling season off the coast of Oregon, USA.

### DOP production

A primary objective of the simulated phytoplankton bloom experiments reported on here was to observe and quantify the response of the DOP pool to perturbations in the nutrient regime, and to decouple potential DOP production from consumption under varied environmental conditions. To achieve this goal, DOP was assayed in (+N) treatments designed to force production of APA, and in (+P) treatments, intended to repress APA, taking advantage of P-regulation of the alkaline phosphatase enzyme, in an effort to maximize and minimize DOP consumption, respectively. While the (+N) treatments allow us to probe the behavior of the phytoplankton community when it is forced to maximally utilize DOP, and the (+P) treatments allow us to examine DOP production under conditions that minimize DOP utilization, Control treatments nominally represent the natural progression that would occur *in situ*.

This novel approach to assaying DOP production carries with it certain constraints that are typical of bottle incubations, in general. For example, removal of grazers may cause shifts in community composition, including both the phytoplankton and heterotrophic microorganism components of the community, which may be accompanied by changes in enzyme activities that could influence patterns of DOP production. In addition, other phosphohydrolytic enzymes, not quantified as part of this study, may be active and may further impact the observed patterns of DOP production (as well as DIP drawdown). Another potentially important consideration is that the bioavailability of the *in situ* DOP pool, as well as the nature of any new DOP produced over the time-course, will potentially affect rates of DOP consumption by hydrolytic breakdown, and therefore impact predictions about DOP production rates. Without knowledge of the composition and bioavailability of the DOP pool, however, it is not possible to make inferences about the impact these factors might have on predicted DOP production rates. In future studies of this kind, it will clearly be valuable to include assays of microbial community composition, particulate phosphorus concentration and composition, as well to obtain compositional information on the DOP pool where possible, in order to constrain uncertainties in mechanisms driving observed trends in P cycling. These additional sorts of data would enhance our ability to map out, more explicitly, the pathways of P uptake, retention (including as luxury polyphosphate stores), and release by cells under different environmental conditions.

The (+N) treatment was designed to evaluate minimum DOP production. In experiments at all stations (+N) treatments resulted in increased chl *a* and APA. DIP was drawn down to undetectable levels by day-5 in (+N) treatments [within the analytical uncertainty of the analysis (±0.02 μM)], implying that DOP utilization was occurring at maximal rates. On the CP-line (+N) treatments yielded lower DOP production rates relative to Controls, as well as increased APA, suggesting that DOP levels were depressed as a consequence of increased hydrolysis of bioavailable DOP substrates. In contrast, DOP production rates for Control and (+N) treatments at site CH-6 were nearly indistinguishable. Examination of ambient nitrate concentrations, which were similar to or lower than the other stations (Wetz and Wheeler, 2005), allowed us to rule out the possibility that high *in situ* nitrate levels might have depressed the APA and DOP production response to added nitrate at CH-6.

The (+P) treatment was designed to evaluate maximum DOP production. In the (+P) treatments there was no systematic increase in chl *a* relative to Controls, APA was similar to Controls, and DIP drawdown was minimal, implying reduced potential hydrolysis of DOP (Dyhrman and Ruttenberg, 2006). This is consistent with the observation of DOP accumulation with DIP addition. One factor that may contribute to the minimal DIP drawdown observed in (+P) treatments is phytoplankton release of short-chain polyphosphates during the incubation; these would be assayed as DIP by our analytical protocol. DOP concentration increased dramatically at all sites in (+P) treatments, suggesting that at high levels of DIP phytoplankton either synthesize more DOP, and/or excrete higher levels of DOP, than they otherwise would retain. Apparent DOP production in the absence of a significant increase in chl *a* in the (+P) treatments could result from enhanced cellular conversion of DIP to DOP, and/or enhanced release of DOP through exudation or excretion, relative to cells in the (+N) treatments. In other words, new biomass is not required to generate DOP if cells produce DOP from internal pools of DIP, and then release that DOP to the medium via excretion, exudation, or lysis. In future work, it would be valuable to assay a more detailed time course, and to assess a combined (+N and +P) treatment to evaluate coupled N-P effects on DOP production.

Application of the two end-member (maximum and minimum) DOP production rates to initial (*in situ*) DOP concentrations observed at each station allows us to predict maximum and minimum DOP concentrations that would have occurred *in situ*, by day-5, if these end-member rates were operant. Contrasting these end-member predictions with the actual DOP concentration that is observed in the Control treatment on day-5 permits us to evaluate which end-member rate most closely approximates the *in situ* rate, and to consider the implications of this result for DOP cycling. The net DOP concentration predicted using the maximum DOP production rate substantially exceeds the observed DOP concentration in the Controls at the end of the 5-day incubation at all sites, which implies that in the presence of excess DIP, which characterized the (+P) treatments, net DOP production is expected to exceed DOP consumption. In contrast, use of the minimum net DOP production rates results in predicted net DOP concentrations that either equal or exceed DOP concentrations observed in Controls by day-5 of the incubations. Specifically, under minimum net DOP production rates, predicted net DOP and observed DOP concentrations for relaxation site CH-6 are indistinguishable, while DOP consumption exceeds net DOP production at all other sites.

The rates of DOP production calculated from these incubation experiments provide a measure of the quantity of DOP produced in a given parcel of water, without consideration of the nature (nutritional status, growth stage) or size of the phytoplankton biomass in that parcel of water. We can use the rates observed in these experiments, and the predictions made using these rates, to speculate upon the magnitude of DOP export offshore. Offshore transport of surface waters occurs during the upwelling season off central and northern Oregon within <1–4 weeks after upwelling (Barth et al., 2005). Thus the day-5 DOP concentrations attained in the Control treatments of our experiments, which ranged between 0.26 and 0.45 μM, could represent the concentration of DOP in waters advected from the Oregon upwelling coast to offshore ecosystems. Depending upon levels of DIN and DIP that are present *in situ*, our incubation experiments suggest that the level of DOP produced could be diminished (in the case of N-enriched waters) or elevated (in the case of P-enriched waters) relative to the *in situ* levels approximated by our Control treatments (Table 4). For example, rivers tend to be enriched in nitrate relative to phosphate; rivers draining coastal Oregon, including the Columbia River, are enriched in both nitrate and ammonium (Wetz et al., 2006). During times of significant riverine input, when relatively DIN-enriched conditions can be realized after strong, DIN-rich freshwater pulses to the coastal ocean, our results suggest that lower net DOP production would result owing to heightened DOP utilization. Conversely, higher net DOP production and export would be expected during times of low freshwater input, when DIN-enrichment should be minimized. On the Oregon coast, where riverine input tends to be seasonal (maximum input during winter/early spring: Castelao and Barth, 2005; Wetz et al., 2006), one might therefore expect seasonal shifts in the magnitude of DOP export from the coastal region to offshore ecosystems.

### Relating patterns of DIP drawdown and DOP production to upwelling and relaxation

Our strategy was to examine results of incubations initiated with waters collected during both upwelling and relaxation conditions in order to evaluate how these different initial conditions might affect DOP production. The status of stations with respect to whether upwelling or relaxation prevailed was defined primarily by temperature. However, other parameters were consistent with this categorization. Higher average DIP concentrations were observed at upwelling sites relative to relaxation sites, which is also consistent with upwelled surface waters. The higher chl *a* at upwelling sites represents growth of phytoplankton biomass in response to upwelling of deeper, nutrient-enriched waters to the surface (e.g., see Small and Menzies, 1981; Landry et al., 1989; Hill and Wheeler, 2002; Ruttenberg and Dyhrman, 2005). The DOP concentrations at all four sites are remarkably similar, despite the substantial differences in physical conditions (e.g., upwelling versus relaxation) and DIP concentrations. Ruttenberg and Dyhrman (2005) found that deep waters at these sites were substantially depleted in DOP relative to surface waters, so the relatively high concentrations of DOP observed in surface waters at all four sites must derive from *in situ* production of DOP.

Characterizing the biogeochemical environment in a dynamic coastal upwelling system is challenging because of the inconstant nature of surface waters, which will display different biogeochemical characteristics depending upon when, during the upwelling – post-upwelling – relaxation – downwelling cycle surface waters are sampled. Based upon surface water temperatures, the surface waters at station CP-1 were sampled sooner after upwelling than station CP-4/5, which was sampled after sufficient time had elapsed for surface water temperatures to warm by 1.4°C relative to those observed at station CP-1. Rates of DOP production in Controls at the two upwelling sites are consistent with this presumed timing, with higher rates observed at the most recently upwelled site (CP-1), which also was characterized by higher *in situ* DIP levels than the upwelling site with warmer surface waters (CP-4/5). Following this reasoning, relaxation sites would be expected to display lower effective DOP production rates, because DOP will be more extensively utilized in the older, warmer, more DIP-depleted waters that characterize relaxation sites. While lower rates were indeed observed in Control treatments at both relaxation sites relative to upwelling sites, the relationship to temperature imperfectly adheres to the pattern just described. In particular, the relaxation station with the warmest surface waters (CP-4) displays a rate more similar to the upwelling sites than does the rate calculated for the other relaxation site (CH-6), which is characterized by surface water temperatures intermediate between the upwelling sites and relaxation site CP-4. In fact, relaxation site CH-6 displays a substantially lower DOP production rate than any of the stations along the CP-line, including the other relaxation site at CP-4.

Geographic location thus appears to be a stronger determinant for rate and extent of DOP production than initial physical (upwelling versus relaxation) conditions. In addition to the patterns for Control treatments just described, incubations of all sites along the CP-line resulted in final (day-5) DOP concentrations in (+N) treatments that are lower than Controls, indicating that DOP consumption exceeded DOP production in these incubations. Incubation of CH-6 surface waters, in contrast, resulted in final (day-5) DOP concentrations in (+N) treatments that are statistically indistinguishable from Controls. These patterns are not driven by the differences in phytoplankton biomass between the upwelling and relaxation experiments, because biomass-normalized rates of DOP-production displayed patterns between stations similar to bulk rates. Consistent with the contrast in DOP production rates along the CP- versus the CH-line just described, both raw and specific APA are higher *in situ* at the three CP sites relative to the CH-line (Table 2), and no systematic difference between either raw or specific APA occurred in upwelling versus relaxation sites. Thus, initial conditions in the Heceta Bank (CP-line) sites are more favorable for DOP hydrolysis than conditions along the CH-line.

The most obvious distinction between the two geographic locations along the Oregon coast that coincide with the CP- and the CH-lines is the bathymetry. The CP-line transects the northern portion of Heceta Bank, a shallow platform characterized by water residence times ≈10× longer than the more northerly portion of the COAST grid where the CH-line is located (Barth et al., 2005; Castelao and Barth, 2005). Ruttenberg and Dyhrman (2005) argued that bathymetrically influenced differences in water residence times were responsible for the negative correlation of *in situ* DIP and APA observed along the CP-line, and the absence of such a relationship along the CH-line. With a longer water residence time on Heceta Bank, the DIP pool can be drawn down to a greater extent, with the result that the DOP pool takes on greater importance for supporting phytoplankton production, as was originally argued by Ruttenberg and Dyhrman (2005). The fact that the balance between DOP production and consumption observed in the incubation experiments also appears to trend with geographical location rather than *in situ* physical (upwelling versus relaxation) conditions suggests that bathymetry, particularly as it affects water residence times, may be a more important constraint on DOP cycling than upwelling/relaxation state.

Studies that have examined relative rates of DOC, DON, and DOP remineralization typically find that N and P are remineralized preferentially to C (e.g., Hopkinson et al., 2002), which is also the pattern observed for POM (e.g., Knauer et al., 1979; Karl et al., 1996). Wetz et al. (2008), however, saw little evidence for preferential degradation of DON relative to DOC in incubations of seawater from the Oregon continental shelf (DOP was not measured in their study). These authors speculate that because the DOC pool in Oregon coastal surface waters appears to be remineralized preferentially to the DON pool, N may be preferentially exported from the system relative to C. While our study was not designed to contrast DOP mineralization relative to DOC and DON, the presence of detectable APA in all samples examined in this and in previous studies of this system (Ruttenberg and Dyhrman, 2005; Dyhrman and Ruttenberg, 2006) suggests that some component of the ester pool is being hydrolyzed (and thus remineralized) *in situ*. It would be valuable in future studies of Oregon coastal waters to examine the relative remineralization rates of DOP relative to DON and DOC in order to validate the finding of a relatively recalcitrant DON pool (Wetz et al., 2008), and to evaluate whether DOP is similarly recalcitrant relative to DOC.

It is interesting to speculate, given the nature of the two bathymetrically distinct regions under study, each with substantially different water residence times, that the composition of the DOP pool in the two regions may also be distinct. One possibility for the lower net DOP production rates observed for all CP-line stations is that the DOP pool in Heceta Bank waters is more bioavailable than that encountered along the CH-line. It is also possible that the phytoplankton communities at the two sites are distinct, with the population residing in Heceta Bank waters predisposed to more efficiently utilizing DOP than the phytoplankton community found on the narrower shelf further north. For example, in coastal populations of phytoplankton there are often higher percentages of dinoflagellates with APA than diatoms (Dyhrman and Ruttenberg, 2006; Mackey et al., 2012), which could drive differences in DOP cycling. In either case, the extent to which DOP is remineralized in the near-shore will affect its relative bioavailability once it is exported offshore, which will have implications for the degree to which it will influence productivity of offshore ecosystems.

## Conclusion

In this study, patterns of DOP production were evaluated by tracking the evolution of DOP during simulated phytoplankton blooms initiated with nutrient amended surface waters, relative to controls, from the Oregon (USA) coastal upwelling system. A particular challenge presented by the resulting data sets was to decouple DOP production from DOP consumption in order to evaluate potential controls on *in situ* DOP production rates. We adopted the approach of estimating maximum [under (+P) conditions] and minimum [under (+N) conditions] DOP production rates, and applied these rates to *in situ* DOP levels to evaluate which end-member rate most closely approximates the *in situ* rate at four stations in this coastal system. Although there are caveats to this approach, this first attempt at approximating DOP production in a coastal upwelling system provides new insight into how DOP production might vary under different environmental conditions. Considerable DOP was produced in (+P) treatments, which suggests that changes in the DIN:DIP ratio of waters could exert profound control over DOP production rates in this and potentially other systems. Patterns of DOP production across the different experiments suggest that bathymetry-driven differences in water residence times can influence DOP cycling, an observation consistent with previous work (Ruttenberg and Dyhrman, 2005). Experimental results provoked a number of intriguing questions, including whether seasonal differences in the dissolved inorganic nutrient regime might influence the inherent bioavailability of DOP produced, as well as impact the magnitude of the DOP available for export to offshore ecosystems. It will be valuable in future studies that build on the strategy for examining DOP production rates adopted here to include parallel tracking of both dissolved and particulate nutrient concentrations in order to more fully map out trends in P cycling, and to strategically site future incubation studies to confirm and refine our understanding of the nature and extent of shelf water residence time control on P cycling and ultimate DOP export off shore.

## Conflict of Interest Statement

The authors declare that the research was conducted in the absence of any commercial or financial relationships that could be construed as a potential conflict of interest.
